# MR Angiography, MR Imaging and Proton MR Spectroscopy *In-Vivo* Assessment of Skeletal Muscle Ischemia in Diabetic Rats

**DOI:** 10.1371/journal.pone.0044752

**Published:** 2012-09-21

**Authors:** Stefano Delli Pizzi, Rosalinda Madonna, Massimo Caulo, Gian Luca Romani, Raffaele De Caterina, Armando Tartaro

**Affiliations:** 1 Department of Neuroscience and Imaging, Institute for Advanced Biomedical Technologies, “G. d'Annunzio University” Foundation, Chieti, Italy; 2 Department of Neuroscience and Imaging, Cardiology Division of Center of Excellence on Aging, University “G. d'Annunzio”, Chieti, Italy; University of Bristol, United Kingdom

## Abstract

To prospectively evaluate the feasibility of using magnetic resonance (MR) techniques for *in-vivo* assessing a rat diabetic model of limb ischemia. Unilateral hind limb ischemia was induced by ligation of the iliac-femoral artery in male streptozotocin-treated and non-diabetic control rats. Four weeks after ligation, rats underwent MR Angiography (MRA), T_1_-weighted and Short Time Inversion Recovery (STIR) sequences and muscle Proton MR Spectroscopy (^1^H-MRS) on both hind limbs. After MR examinations, immunoblotting and immunofluorescence analysis were performed. MRA showed a signal void due to flow discontinuation distal to the artery ligation. T_1_-weighted and STIR images showed, respectively, the presence of tissue swelling (p = 0.018 for non-diabetic; p = 0.027 for diabetic rats) and signal hyperintensity in tissue affected by occlusion. Mean total creatine/water for the occluded limb was significantly lower than for the non-occluded limbs in both non-diabetic (5.46×10^−4^ vs 1.14×10^−3^, p = 0.028) and diabetic rats (1.37×10^−4^ vs 1.10×10^−3^; p = 0.018). MR Imaging and ^1^H-MRS changes were more pronounced in diabetic than in non-diabetic occluded limbs (p = 0.032). MR findings were confirmed by using histological findings. Combined MR techniques can be used to demonstrate the presence of structural and metabolic changes produced by iliac-femoral artery occlusion in rat diabetic model of limb ischemia.

## Introduction

In humans, lower limb ischemia leads to progressive structural and functional deterioration of tissue, eventually leading to necrosis and amputation [1]. Animal models using femoral artery ligation were developed to mimic unilateral lower limb ischemia, permitting the assessment of concurrent morphologic and metabolic processes [2].

Epidemiological evidence indicates a relationship between diabetes mellitus and prevalence/severity of peripheral arterial disease [3]. Hyperglycemia leads to the development of myopathy and microangiopathy, determining myocyte loss, endothelial dysfunction and fibrosis [3], [4]. Fibrosis and endothelial dysfunction may worsen muscular blood flow reserve, possibly altering energy metabolism and organ dysfunction. These abnormalities lead to limited exercise tolerance and greater vulnerability to ischemia. Hyperglycemia can be pharmacologically induced in rodents by administering streptozotocin (STZ), which selectively destroys pancreatic beta-cells, therefore reproducing type-1 diabetes [5]. Studies on STZ-treated diabetic mice have shown that a superimposed ischemia induced by an arterial occlusion can further worsen hyperglycemia-induced apoptosis of endothelial cells [6].

Although currently histological studies [7] are considered the reference standard to evaluate consequences of ischemia in animal models [8], noninvasive techniques such as magnetic resonance (MR) may be a valuable tool for *in-vivo* assessment of the physiopathology of peripheral tissue disease and for monitoring the efficacy of novel pharmacological compounds over time.

In rabbit models of hind limb ischemia, MR Angiography (MRA) was reported as a potential tool for the non-invasive assessment of vessel occlusion [9]–[11]. MR Imaging (MRI) is able to identify the site and to determine the severity of ischemic tissue injury. T_1_-weighted images (T_1_-WI) can show hind limb morphology and soft tissue swelling after ischemic injury [12]. Short Time Inversion Recovery (STIR) sequence can improve the detection of “oedema-like” tissue modifications related to muscle infarction [13]–[15]. Proton MR spectroscopy (^1^H-MRS) offers interesting metabolic information about skeletal muscle [16]–[20] and the effects of diabetes [21], [22] on it. Particularly, tCr is a marker of cellular energetic metabolism and its level was reduced in previous studies concerning infracted myocardium [23].

The aim of this study was to prospectively evaluate the feasibility of using magnetic resonance (MR) techniques for *in-vivo* assessment of a rat diabetic model of limb ischemia. To validate our MR protocol, we compared MR outcomes with immunoblotting and immunofluorescence results.

## Methods

### Animal Care

This prospective study was approved by our Institutional Ethics Committee for animal research and conformed to the “Principles of Laboratory Animal Care” formulated by the National Society for Medical Research and “Guide for the Care and Use of Laboratory Animals” [NIH Publication 86-23, received 1985].

### Materials

STZ was obtained from MP Biomedicals (Solon, OH). All other chemicals were purchased from Sigma-Aldrich (St. Louis, MO).

### Multiple low-dose streptozotocin-induced hyperglycemia

Six week-old male Sprague Dawley rats (n = 13, 350–400 g) were randomly assigned to two groups: diabetic (*n* = 7) and control (*n* = 6). Rats received a daily tail vein injection of either 50 mg/kg STZ dissolved in trisodium citrate buffer (1 mL/kg of 0.01 M, pH 4.5; Sigma) or buffer alone, respectively, for six consecutive days. Blood glucose was monitored weekly over the following 30 days using Ascensia Elite XL one-touch blood glucometer (Bayer). Animals were considered diabetic when their fasting blood glucose level was ≥400 mg/dL and confirmed by a second sample taken within 24 hours.

### Unilateral Hind Limb Ischemia

Eight weeks after diabetes induction, unilateral limb ischemia protocol was performed. Before surgery, rats were anesthetized with a mixture of oxygen and halothane (2.5%), sodium pentobarbital (intra-peritoneally, i.p., 50 mg/Kg) and sodium heparin (1000 U/Kg). The common right iliac-femoral artery was ligated according to reported methods [2], [24]. The overlying skin was closed. Animals received standard postoperative care.

### MR experiment

**Figure 1:**
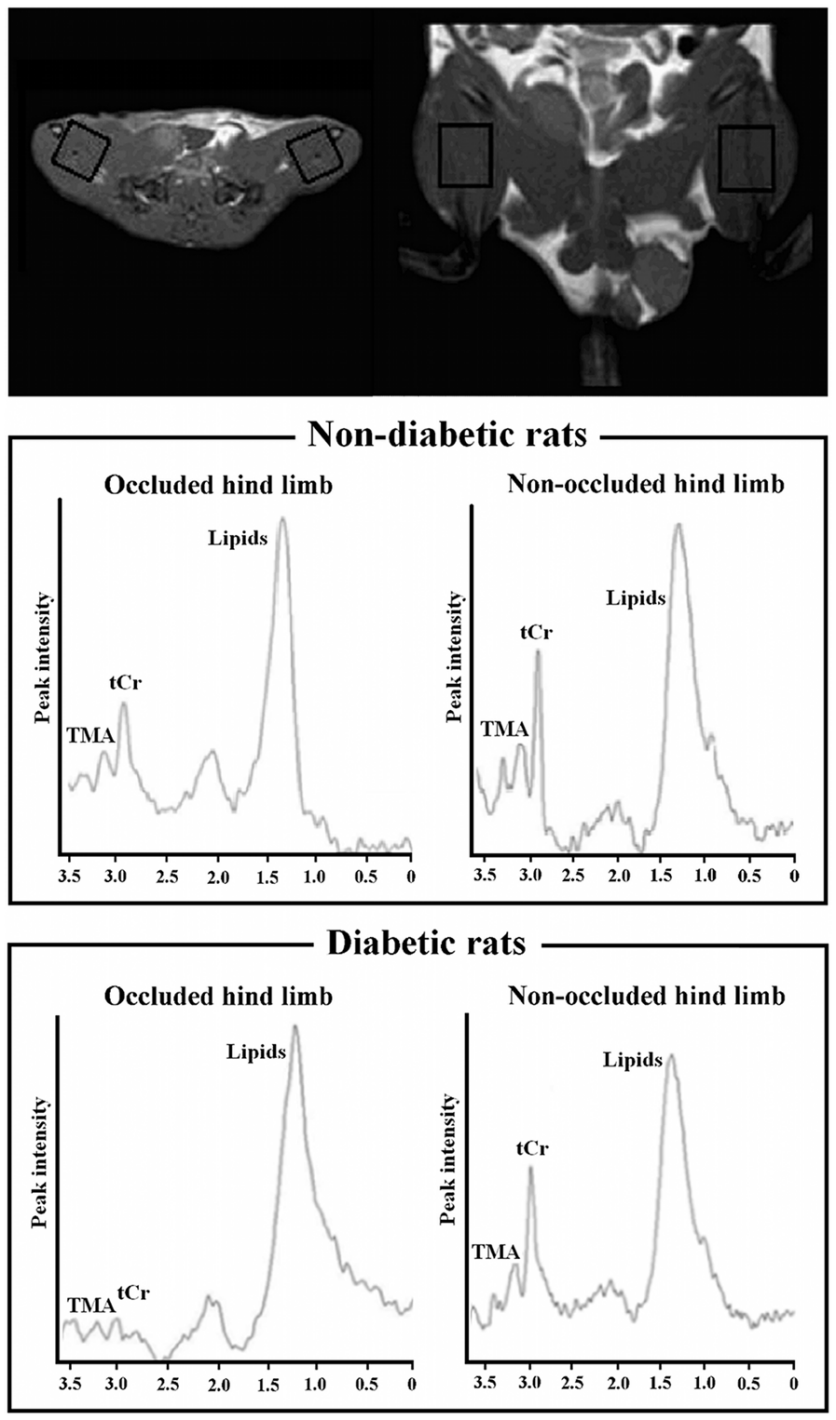
Representative ^1^H-MR spectra. A voxel of 12×12×15 mm^3^ was places on right ischemic and left non-ischemic limbs. The tCr peak was observed at 3.03 ppm, trimethyl ammonium-containing compounds (TMA) at 3.21 ppm and lipid resonance at 0.9–1.4 ppm. The smaller tCr peak observed in the ischemic limbs is indicative of ischemic damage in the muscle.

All animals underwent MR examination four weeks after the right iliac-femoral artery ligation. Before each MR acquisition, rats were anesthetized by the same protocol used during surgery. Measurements were performed with a 3 T scanner (Philips Medical System, Best, the Netherlands), equipped with a sense flex surface coil. The animals were placed in a supine position and their hind limbs were placed between the two coil rings. Axial, coronal and sagittal images of hind limbs were performed by T_1_-W spin-echo sequence (TR/TE = 742/17 ms, slice thickness 2.5 mm, matrix size 156×162, 12 slices, and FOV = 100×100 mm). STIR in axial orientation was acquired (TR/TE/TI = 3393/30/160 ms, slice thickness 2.5 mm, matrix size 148×144, 18 slices, and FOV = 80×130 mm). MRA was performed with TOF 2D sequence: TR/TE = 18/3.5 ms, slice thickness 2 mm, matrix size 150×50, 60 slices, and FOV = 150×50 mm. A single voxel (12×12×15 mm^3^) was located on the adductor and semimembranous muscles of each hind limb (**Figure 1**) and a PRESS sequence (TR/TE = 2000/50 ms, 16-step phase-cycle, averaged for 192 scans and 2048 points with 2.000 Hz spectral width) with and without CHESS water suppression was acquired.

### MRI data assessment

**Figure 2:**
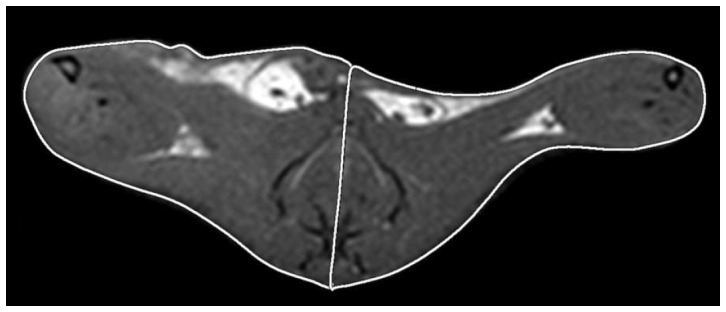
Occluded and non-occluded hind limbs on T_1_-W images. T_1_-W axial images were used under double blinding to manually draw and measure the areas of right (occluded) and left (non-occluded) hind limbs. A representative image shows white boundaries that delimit the measured areas in each rat. These two regions of interest (ROI) were divided by a line passing from anterior/ventral to posterior/dorsal sides, crossing perpendicularly the vertebral column. Subsequently, left and right ROI areas were calculated using MR work station, normalized respect to the total limbs area (left limb area+right limb area) and expressed such as size index [ROI size index = ROI area/total limbs area)]. To assess the difference between occluded and non-occluded limbs, a quantitative “swelling index” was calculated for each animal [Swelling Index = (Area of right limb−Area of left limb)/(Area of right limb+Area of left limb)].

All MRI data were analyzed on a Philips MR work station. MRA images were visually checked for the presence of a signal void due to flow discontinuation distal to the right common iliac-femoral artery. T_1_-W and STIR axial images were used under double blinding to respectively measure the areas of the occluded (right) and non-occluded (left) hind limb and to identify the presence of signal hyperintensity related to oedema. On T_1_-W images, the boundaries of occluded and non-occluded hind limb were manually drawn (**Figure 2**). For each rat, the areas measured from the occluded and non-occluded hind limb were normalized to the total area (right limb area+left limb area) and expressed as “size index” [limb of interest area/(right limb area+left limb area)]. In order to assess the difference between occluded and non-occluded limbs, a quantitative “swelling index” was calculated for each animal [swelling index = (right limb area−left limb area)/(right limb area+left limb area)].

Visual evaluation of STIR images was performed independently by two radiologists. The “oedema-like” extension detected as signal hyperintensity in STIR images, was scored on a three-grade scale [25]: not detectable (grade 0), “oedema-like” extension ≤50% than whole limb (grade 1) and “oedema-like” extension >50% than whole limb (grade 2). In order to resolve discordant STIR scoring, consensus reading agreement between the two radiologists was acquired.

### MRS data processing

All spectra were analyzed by using the AMARES algorithm within jMRUI [26]. Water suppressed spectra were filtered for removal of residual water by using the HLSVD method [27]. Autophasing and baseline correction were applied. From each unsuppressed spectra, the area of the water peak was calculated by the same protocol to establish a reference signal for use as an internal standard [28]. All non-water signals were removed from the unsuppressed free-induction decays by using the HLSVD method.

### Immunoblotting

Total proteins from ischemic and non-ischemic skeletal muscle tissues were isolated in ice-cold radio-immuno precipitation assay (RIPA) buffer, separated under reducing conditions, and electro-blotted to PVDF membranes (Immobilon-P, Millipore Bedford, MA). Western blot analysis of type III collagen and α-sarcomeric actinin was performed by incubating the membranes overnight at 4°C with the following primary antibodies: (1) monoclonal rabbit anti- α-sarcomeric actinin antibody (Sigma Aldrich, St Louis CA); (2) monoclonal goat anti-collagen type III antibody (Sigma). Blots were incubated with horseradish peroxidase-coupled secondary antibodies, washed and developed by using a SuperSignal West Pico Chemiluminescent Substrate Kit (Pierce, Rockford, IL). The intensity of each immunoreactive protein band was measured by densitometry. To verify equal loading of proteins, membranes were stripped and re-probed with monoclonal anti-beta actin antibody (Sigma).

### Immunofluorescence microscopy

The effect of femoral artery occlusion on neovascularization was assessed in 5 µm-thick frozen sections taken from the adductor and semimembranous muscles from both the ischemic and non-ischemic limbs. Sections in optimal cutting temperature (OCT) compound were fixed with 4% paraformaldehyde, permeabilized, blocked for 30 min in phosphate-buffered saline (PBS) containing 1% bovine serum albumin, and incubated with a fluorescein-conjugated anti-von Willebrand factor monoclonal antibody (vWF, Sigma) at 4°C for 1 h. Non-immune IgG was used as the isotype control (Becton, Dickinson and Co.). The sections were washed, mounted in glycerol mounting medium (VectaShield, Vector Labs) and viewed under a fluorescence microscope. The number of capillary vessels were counted. Five fields from the 3 different muscle samples of each animal were randomly selected for the capillary density analysis. The data are presented as number of capillary/muscle area ratio.

### Statistical Analysis

Statistical analysis was performed using SPSS 15.0. The Wilcoxon test was used to compare differences within diabetic and non-diabetic group (occluded vs. non-occluded limbs). Mann-Whitney test was used to compare differences of occluded and non-occluded limbs in diabetic as respect to non-diabetic group (occluded in non-diabetic vs. occluded in diabetic; non-occluded in non-diabetic vs non-occluded in diabetic). Pearson's correlation investigated the potential relation between the T_1_-W and between MRS and histological outcomes (sarcomeric α-actinin, type III collagen, capillary density) respectively. Spearman's correlation described the relation between STIR “oedema-like” score and histological outcomes.

## Results

No rats were lost during diabetes induction, hind limb ischemia and MR protocols.

### Diabetes induction

Seventy-two hours after STZ administration a rise in blood glucose was observed in all rats. A stable induction of diabetes was obtained during the first week in all rats.

### TOF MR Angiography

**Figure 3:**
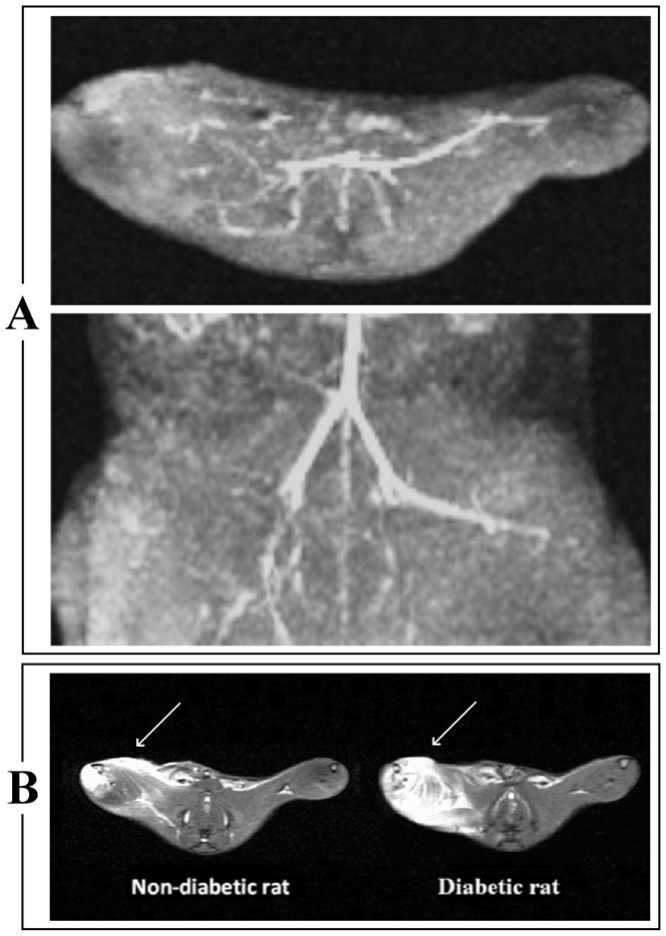
Representative MR Angiography (MRA) and Short Time Inversion Recovery (STIR) images. **Panel A:** MRA shows a signal void due to discontinuation of flow in the right common iliac-femoral artery. **Panel B:** Axial STIR images shows an “oedema-like” hyperintense signal (indicated by white arrows) on the occluded (right) muscle in both non-diabetic and diabetic rat. This signal changes is more extensive in the diabetic rat.

MRA showed a signal void due to flow discontinuation distal to the right common iliac-femoral artery in all rats (**Figure 3A**).

### MR Imaging

**Figure 4:**
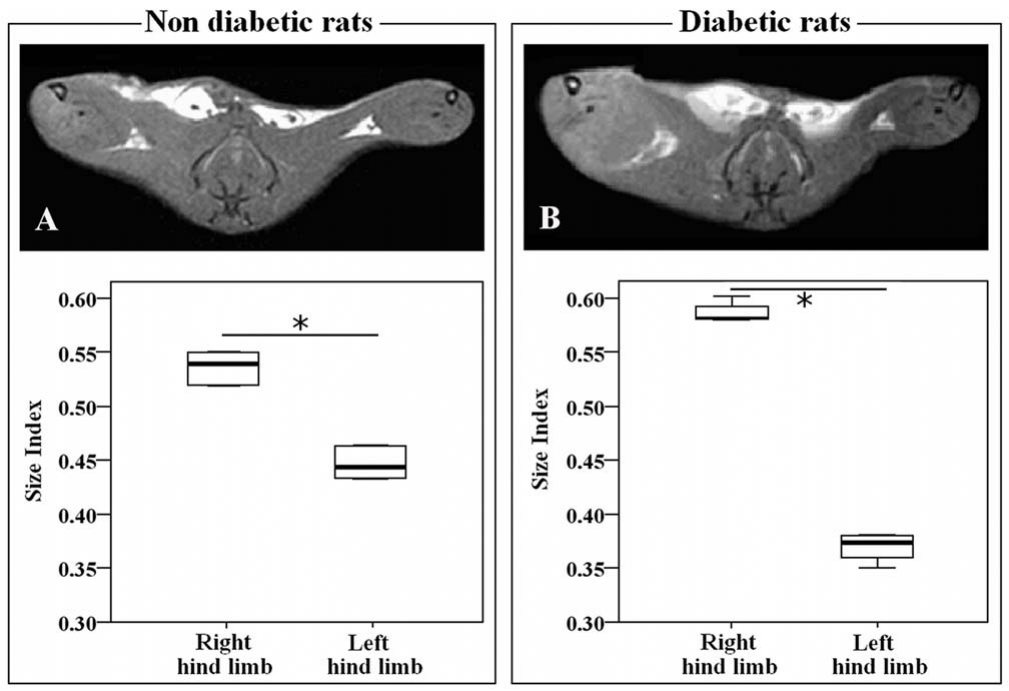
Representative T_1_-W images of the occluded and non-occluded hind limbs and morphometric outcomes. Left and right panels are referred to non-diabetic and a diabetic rat, respectively. Axial T_1_-W images show increase in soft tissue size due to regional swelling after right common iliac-femoral artery ligation in both non-diabetic (A) and diabetic (B) rat. Box and Whiskers Plots show the distribution of size indices of occluded [size index = right limb area/(right limb area+left limb area)] and of non-occluded limb [size index = left limb area/(right limb area+left limb area)]. Statistically significant differences were noted comparing the size index of occluded and non-occluded limb both in diabetic (n = 7) and non-diabetic (n = 6) rats. Significance level: * p<0.05. Box and Whiskers plot legend: the bottom and top of the box represent respectively the lower and upper quartiles; the bold band is the median; the ends of the whiskers represent the minimum and the maximum value.

The STIR sequence (**Figure 3B**) showed an “oedema-like” hyperintense signal on occlusion of right limbs in all rats. Using the three-grade scale, there was a marked difference between diabetic and non-diabetic rats. In all diabetic rats, “oedema-like” hyperintense signal extension was >50% than total limb dimension (grade 2). Differently, in all non-diabetic rats, “oedema-like” hyperintense signal extension was ≤50% than total limb dimension (grade 1). T_1_-W images show an increase in soft tissue size due to regional swelling after the right common iliac-femoral artery ligation in both non-diabetic (mean size index for right limbs = 0.54 vs mean size index for left limb = 0.44; p = 0.018) and diabetic rats (mean size index for right limbs = 0.58 vs mean size index mean size indices for left limbs = 0.38; p = 0.027) (**Figure 4**). The swelling index was higher in STZ-treated rats (mean swelling index for the diabetic group = 0.10; for the non-diabetic group = 0.14; p = 0.026).

### ^1^H MR Spectroscopy

**Figure 5:**
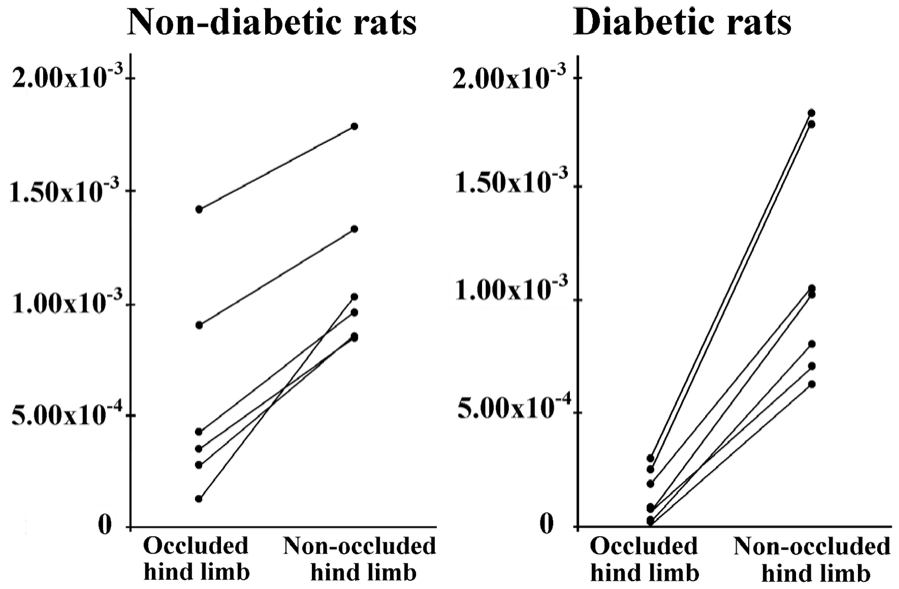
Individual values of tCr/water in each limb. Individual values of tCr/water in the occluded and non-occluded limbs for each non-diabetic (left side) and diabetic (right side) rat (joined by the continuous lines).

Mean tCr/water for the occluded (right) limb were significantly lower than for the non-occluded (left) limbs in both non-diabetic (5.46×10^−4^ vs 1.14×10^−3^; p = 0.028) and diabetic rats (1.37×10^−4^ vs 1.10×10^−3^; p = 0.018) (**Figures 5** **and** **6**). Values of tCr/water for right limbs were significantly lower in diabetic compared with non-diabetic (5.46×10^−4^ vs 1.37×10^−4^; p = 0.046) rats. Values of tCr/water for non-occluded limbs in non-diabetic and diabetic rats did not show significant differences, although the mean value in diabetic rats was lower than in non-diabetic rats (1.10×10^−3^ vs 1.14×10^−3^; p>0.1). The non-occluded-to-occluded differences between diabetic and non-diabetic rats showed a lower energetic metabolism in STZ-treated rats (5.91×10^−4^ vs 9.67×10^−4^; p = 0.032).

**Figure 6:**
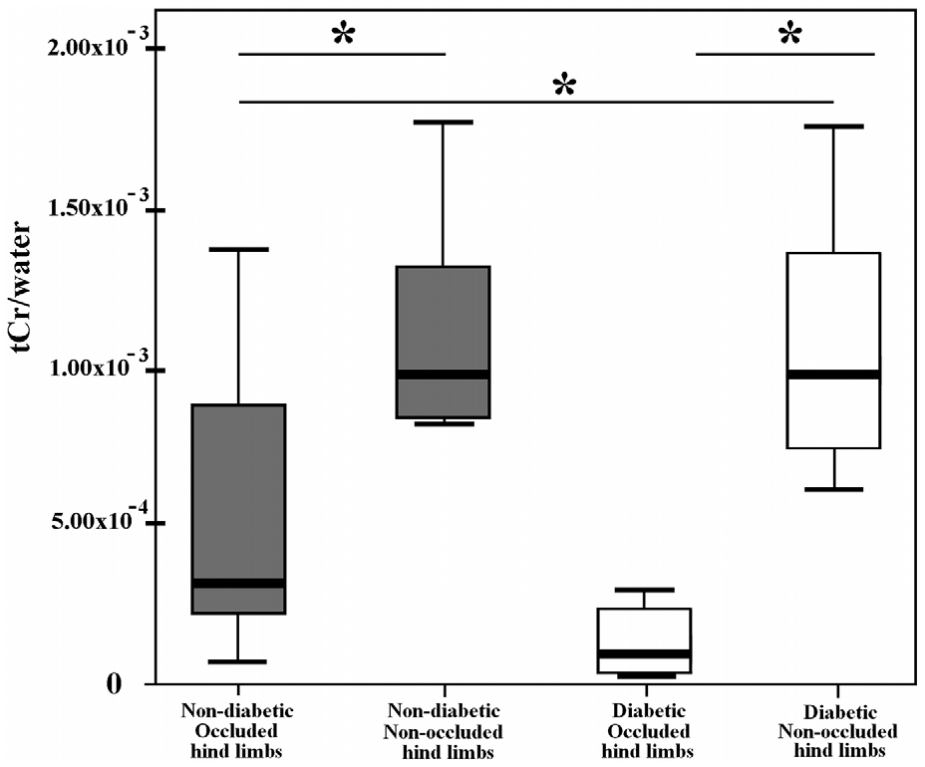
Box and Whiskers plot depicting tCr/water levels between occluded and non-occluded limbs for each groups. Values of tCr/water for occluded limb were significantly lower than non-occluded limbs in both non-diabetic (n = 6, * p<0.05) and in diabetic rats (n = 7, * p<0.05). Values of tCr/water occluded limbs were significantly lower in diabetic compared with non-diabetic (* p<0.05).

### Histological evaluation of capillary and arteriole density, and expression of sarcomeric α-actinin and type III collagen

**Figure 7:**
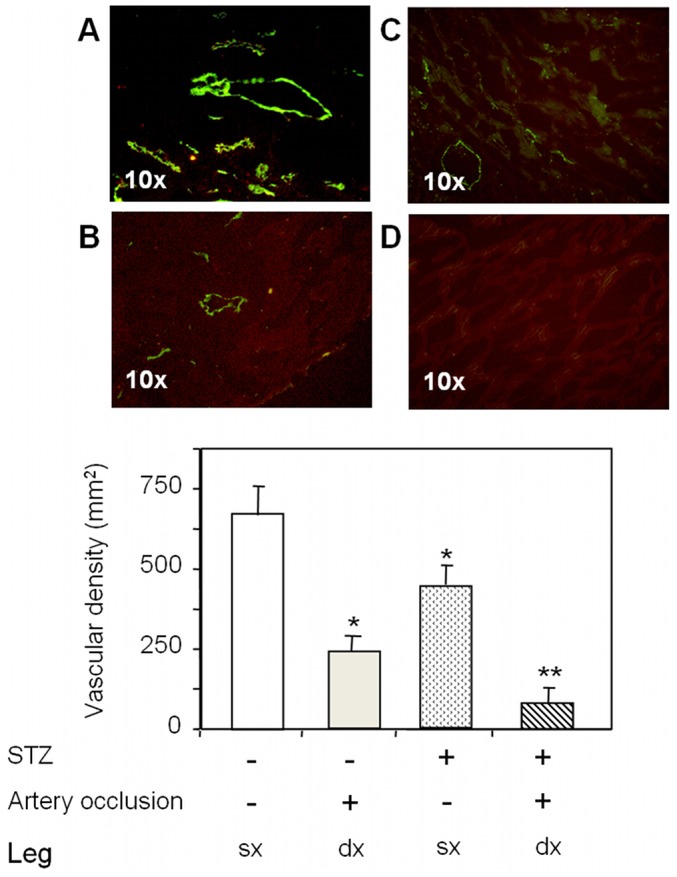
Immunofluorescence analysis. **Panels A, B:** Representative sections of skeletal muscle tissues of non-diabetic rats, taken from non-occluded limbs **(panel A),** occluded limbs **(Panel B),** stained with fluorescein-conjugated anti-von Willebrand factor antibody. **Panels C, D:** Representative sections of skeletal muscle tissues of diabetic rats, taken from non-occluded limbs **(panel C)** and occluded limbs **(panel D)**, stained with fluorescein-conjugated anti-von Willebrand factor antibody. Vascular density was significantly lower in limbs with femoral artery ligation and diabetes. Legend: STZ, streptozotocin-treated rats. Values are mean ± SD. N = 6 for non-diabetic group, N = 7 for diabetic group.

**Figure 8:**
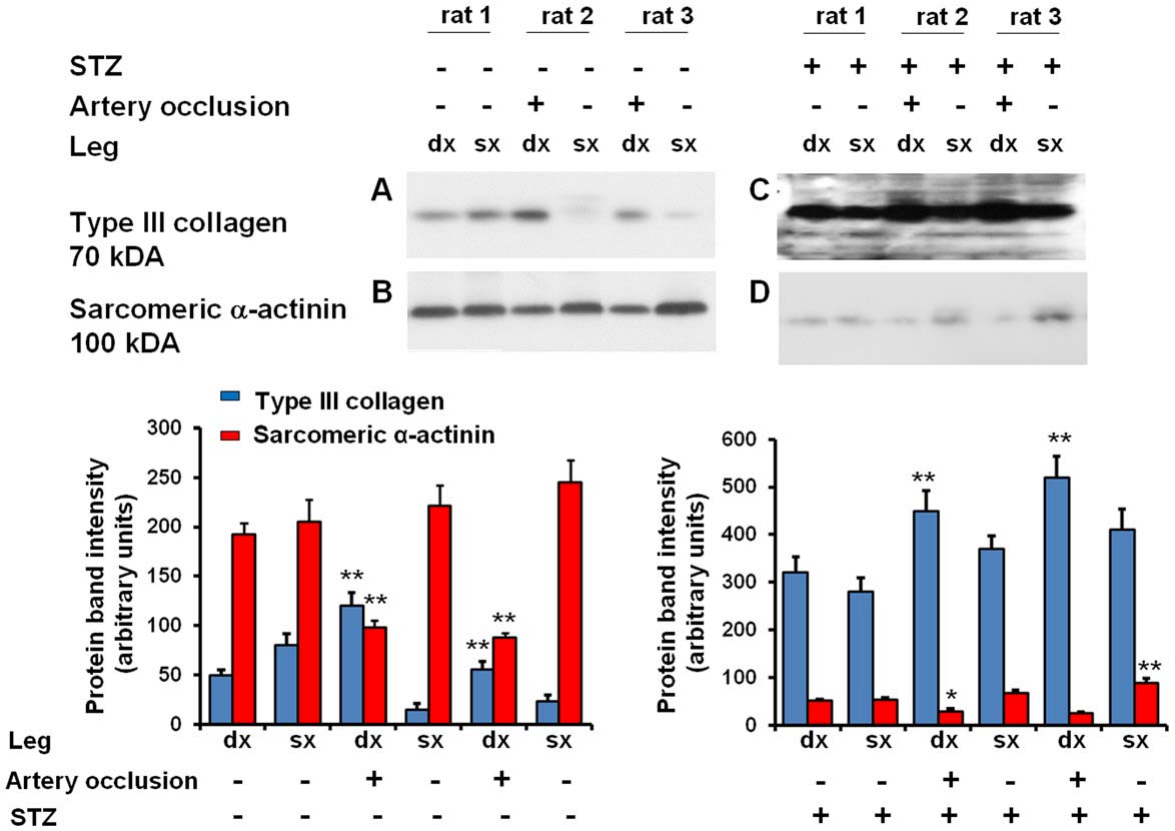
Immunoblotting analysis. **Panels A and B:** Representative immunoblotting (upper panels) and densitometric quantification (data normalized for GAPDH, lower panels) of sarcomeric α-actinin and type III collagen expression in non-occluded limbs (**panel A**) or occluded limbs (**panel B**) from non-diabetic rats. **Panel C and D:** Representative immunoblotting of sarcomeric α-actinin and type III collagen expression in non-occluded limbs **(panel C)** or occluded limbs **(panel D)** from diabetic rats. The blots show increased expression of type III collagen (upper panels) and decreased expression of sarcomeric α-actinin (lower panels). The same blots reprobed with an anti-GAPDH antibody were used as controls for equal loading (not shown). Values are mean ± SD of three independent experiments, with n = 3 rats for each group. * p<0.05 and ** p<0.05 vs normal (non-occluded hind limb). Legend: STZ, streptozotocin-treated rats.

The capillary (**Figure 6 A and B**) and arteriole density (**Table 1**) in the occluded limbs was significantly lower as compared with normal limbs (p<0.01 vs non-diabetic non-occluded limbs) (**Figure 7 A and B**). Protein levels of type III collagen increased and sarcomeric α-actinin decreased in the ischemic tissue of non-diabetic rats (**Figure 8 A and B**). Consistent with the data of MRI and ^1^H MRS, diabetes was associated with a further decrease of capillary density (p<0.05 vs non-diabetic occluded limbs) (**Figure 7 C and D**), decreased levels of sarcomeric α-actinin (**Figure 8 D**), and increased expression of type III collagen (**Figure 8 C**) in the ischemic skeletal muscles of diabetic rats compared with non-diabetic rats.

**Table 1:** Quantitative analysis of arteriole density in the limbs of diabetic and non-diabetic rats.

	Arterioles (vessels/mm^2^)	
	Non-diabetic rats	Diabetic rats
**No ligation (left leg)**	14±2	5±2
**Ligation (right leg)**	7±4*	1±3*

Quantitative analysis of arteriole density from non-ischemic limbs or limbs with femoral artery ligation of diabetic (n = 7) and non-diabetic (n = 6) rats.

Data are presented as mean ± SD,

* p<0.05, versus non-ischemic limbs.

A significant correlation was found between MR outcomes and histological results (**Table 2**).

**Table 2:** Relation between the T_1_W and between MRS and histological outcomes respectively.

	Type III collagen	Sarcomeric α-actinin	Vascular density
**T_1_-WI TSE**	r = 0.32, p = 0.32^b^	r = −0.61, p = 0.04^b^	r = −0.68, p = 0.02^b^
**MRS**	r = 0.22, p = −0.50^a^	r = 0.63, p = 0.03^a^	r = 0.82, p = 0.01^a^
**STIR**	r = 0.58, p = 0.04^b^	r = −0.61, p = 0.04^b^	r = −0.56, p = 0.05^b^

a,b for Pearson's and Spearman's correlation respectively.

*Abbreviations*: T_1_-WI TSE = T_1_-weighted Image Turbo-Spin-Echo; STIR = Short Time Inversion Recovery sequences; ^1^H-MRS = Proton MR Spectroscopy (^1^H-MRS).

## Discussion

Magnetic resonance (MR) techniques have recently shown enormous potential for assessing *in-vivo* and non-invasively, the pathophysiological aspects and the treatment response in small animal models of disease [29]–[31]. Differently from invasive and post-mortem methods, MR-based protocols are also able to longitudinally investigate the disease progression and the therapeutic effects over time, taking into account the inter-individual features. However, the validation of the MR protocol is still an issue that needs to be addressed. In this context, we evaluated the feasibility of using MR techniques for *in-vivo* assessment of a rat diabetic model of limb ischemia. To validate our MR protocol, we compared MRI and MRS outcomes with immunoblotting and immunofluorescence results.

MRA confirmed that the occlusion was effective in all rats, showing a signal void due to the flow discontinuation in the right common iliac-femoral artery consistent with vessel ligation.

MRI is able to detect structural changes in specific ischemic areas. Particularly, an asymmetric profile order in STIR sequences was used to provide an independent parameter selection of TE, echo spacing and turbo factor. MRI allows to select relatively short TE with a relative large turbo factor and, using full scan without image blurring, to improve the signal-to-noise ratio. A short TE reduces the muscle signal degradation [32], helping to provide anatomical details about oedema size and its localization. In agreement with MRI studies on the animal model of limb ischemia [33], [34], we showed the presence of tissue swelling and signal hyperintensity in the right hind limb of all rats who had undergone femoral artery ligation.

Several animal and human MRS studies have investigated the metabolic effect of ischemia on skeletal muscle. ^31^P-MRS studies have shown a reduction of energy metabolism in the ischemic muscle [33]–[37]. Since unphosphorylated creatine (Cr) is inaccessible with ^31^P-MRS, ^1^H-MRS can be a reliable tool for expressing the Cr and PCr contributions as a percent of tCr. Previous studies on ischemic myocardium [23] have reported a reduction of tCr in infarcted regions. In this study, ^1^H-MRS we assessed tCr/water changes, showing a reduction of muscle energy metabolites in occluded versus non-occluded hind limbs, as well as in diabetic versus non-diabetic rats.

MR results are in accordance with immunoblotting and immunofluorescence analysis. We found a reduction of sarcomeric α-actinin and capillary density and increasedtype III collagen in occluded limbs compared with normal limbs, both in diabetic and non-diabetic rats. Type III collagen has been shown to correlate with post-ischemic fibrosis [38]. Sarcomeric α-actinin is an important structural component of the Z-line in skeletal muscle. It maintains the sarcomeric integrity and interacts with a variety of structural, signalling and metabolic proteins [39], [40]. A progressive alteration of α-actinin in skeletal muscle has been shown in an experimental rabbit model of ischemia [41].

Studies have also reported an important correlation between diabetes and peripheral vascular disease. Diabetes induces fibrosis and thickening of the microvascular basal lamina, with subsequent reduction of blood flow reserve and changes in energy metabolism with organ dysfunction [42]. Studies on experimental models of hind limb ischemia in rodents reported significant impairment in post-ischemic hind limb flow recovery in STZ-induced type-1- diabetic mice, as well as in the Lepr db/db mouse model of type-2 diabetes [43], [44].

In agreement with immunoblotting and immunofluorescence results, *in-vivo* MRI and MRS were able to demonstrate more pronounced tissue damage in diabetic than in non-diabetic rats. T_1_-W and STIR sequences showed greater swelling and more evident signal hyperintensity in diabetic than in non-diabetic occluded limbs. Despite the histological analysis showed a significant lower capillary density in diabetic compared with non-diabetic rats, muscle oedema observed on MR images was significantly more pronounced in the diabetic group. The two findings are not necessarily in contradiction, since oedema, as in most cases, may occur without increased capillary density when the permeability functions of the capillaries (“leakiness”) are altered. The larger extent of ischemia could be related to diabetic micro- and macro- angiopathies. In this context, microangiopathy alters capillary permeability [45], [46] and also accelerates atherosclerosis, reducing collateral vessel compensation [46]. Furthermore, oedema determines a mechanical compression of the microcirculation, providing an additional potential explanation as to why the diabetic group shows a lower capillary density in our study. Muscle oedema occurring in diabetes may have additional explanations which can conceivably be related to induced water channel dysfunction(s). Aquaporins (AQPs) are a family of 10 different water-specific, membrane-channel proteins expressed in diverse tissues [47]. Recently a role of AQP1, 4, 8 and 9 in the pathophysiology of myocardial oedema in reperfused swine hearts has been shown [48]. AQP1, which is specifically and strongly expressed in most microvascular endothelial cells outside the brain, is induced by high glucose-induced hyperosmolarity and plays a role in the glucotoxicity-mediated vascular injury [47], [49], [50]. We may therefore hypothesize that the hyperosmotic induction of AQP1 might modulate vascular damage and therefore contribute to the increased oedema here observed in diabetic rats.

^1^H-MRS showed that muscle energy metabolism was reduced in diabetic, rather than in non-diabetic occluded limbs compared with non-occluded control limbs. ^1^H-MRS did not show significant differences in diabetic and non-diabetic non-occluded limbs. However the differences between occluded and non-occluded limbs were higher in diabetic compared with non-diabetic rats.

Although clinical MR system has been widely used for applications on small animals [30], [51], the use of a dedicated high-field scanner can be more advantageous. A slice thickness of 2–2.5 mm and a MRS voxel size of 12×12×15 mm^3^ are able to provide a sufficient signal to noise ratio, but increased total duration of our MR protocol to 40 minutes. In as much as the 3T system provides a better signal to noise ratio respect to 1.5 T, human studies describe disadvantages related to magnetic field dishomogeneity and the dielectric effect [52]. In animal studies, this issue is partially resolved because the field of view is very little and the magnetic field dishomogeneity and dielectric effect are less relevant respect to human acquisition. Another limitation of this study is that with the current sample size, correlation analyses must be interpreted with caution.

An STZ-based model was chosen to assess precisely the hyperglycemic component of diabetes. Despite being a partial model of diabetes type-2 (hyperinsulinemic components of diabetes are missing, but insulin resistance is induced by hyperglycemia), it allows an assessment of the effects of hyperglycemia without the admixture of the effects resulting from hyperinsulinemia. The multiple doses of STZ were administrated to obtain a consistent and sustained hyperglycemia. This treatment is able to prevent no-drug response and the drug-resistance and to avoid the rebound effect of the survived functional beta cells.

In conclusion, our study demonstrates that a combination of MRI, MRA and ^1^H-MRS techniques are suitable to investigate *in-vivo* metabolic and structural alterations induced by hind limb ischemia in a rat diabetic model of limb ischemia. Compared to histological data, all changes induced by arterial occlusion were clearly detected by using our MR-scan protocol.

This scan appears to be promising in preclinical/translational research for evaluating the efficacy of novel pharmacological, as well as novel gene- and cell-based strategies.
